# Multi-parameters of Magnetic Resonance Imaging to Estimate Ischemia-Reperfusion Injury after Stroke in Hyperglycemic Rats

**DOI:** 10.1038/s41598-019-39263-6

**Published:** 2019-02-27

**Authors:** Wei-yuan Huang, Gang Wu, Shan-xi Guo, Dao-ying Geng, Jian-jun Li, Kai Yang

**Affiliations:** 1 Department of Radiology, Hainan General Hospital, Haikou, 570311 China; 2 Department of Radiotherapy, Hainan General Hospital, Haikou, 570311 China; 3 Huashan Hospital, Fudan University, Shanghai, 200040 China

## Abstract

The aim of the study is to verify the effect of hyperglycemia on ischemia-reperfusion injury and to explore the feasibility of noninvasive observation of ischemic-reperfusion injury in hyperglycemic ischemic stroke by MRI technique. According to the duration of ischemia and blood glucose levels, 40 rats were divided into hyperglycemic ischemic 2-hr (H-I2h), hyperglycemic ischemic 6-hr (H-I6h), non- hyperglycemic ischemic 2-hr (NH-I2h), and non- hyperglycemic ischemic 6-hr (NH-I6h) groups. T2W imaging, DW imaging, T2 mapping, T2* mapping, DCE, and T1 mapping after enhancement sequences were acquired before reperfusion and approximately 3-hr after reperfusion. ADC, T1, T2, T2*, and K^trans^ values of ischemic lesion were obtained in different groups. After reperfusion, the variation of ADC values showed no significant difference between groups with diabetes and groups without diabetes and between different recanalization time-points (2-hr vs 6-hr). After reperfusion, T2, T2*, and K^trans^ values increased in different degrees in all four groups. Only the T1 value decreased in all groups. The change of all parameters in groups with hyperglycemia was more obvious than that in groups without hyperglycemia and was more obvious in groups with H-I6h versus those with H-I2h. This study confirms that hyperglycemia aggravates ischemia-reperfusion injury and may be an important risk factor for the prognosis of ischemic stroke. The K^trans^ values should be noninvasive imaging indicators to monitor blood brain barrier permeability and ischemic-reperfusion injury in ischemic stroke.

## Introduction

Hyperglycemia is an independent risk factor of ischemic stroke and can significantly increase stroke incidence and recurrence rate^1,2^. The incidence of ischemic stroke in diabetic patients is 2 to 6 times that of people without diabetes^3^. Clinical and experimental results have demonstrated that diabetes mellitus also increases infarction volume, hemorrhagic transformation (HT) incidence rate, and long-term mortality from stroke and worsens the overall neurologic outcomes that occur after stroke^4–6^.

Hyperglycemia exacerbates ischemic penumbra tissue damage caused by insufficient perfusion. It also causes the accumulation of lactic acid and intracellular acidosis in the ischemic brain tissue, resulting in a more severe neuro-inflammatory response^7^. Hyperglycemia induces a variety of biochemical changes within cerebral vasculature endothelial cells. Hyperglycemia initiates a cascade of events that leads to vascular endothelial cell dysfunction and, finally, increased vascular permeability, especially after recanalization or reperfusion^8,9^.

Previous experimental studies have confirmed that hyperglycemia exacerbates the blood brain barrier (BBB) damage and ischemia-reperfusion injury^10^. Increased BBB permeability with MRI was also detected in patients with type-2 diabetes (T2DM) or white matter hyperintensities^11^. However, no quantitative imaging parameters were used to evaluate BBB permeability dynamically. To the best of our knowledge, use of a noninvasive imaging modality to estimate ischemia-reperfusion injury after stroke in hyperglycemic rats has not been reported. Our research group has been devoted to observing the ischemia-reperfusion injury using MRI. Our earlier studies have confirmed that dynamic contrast enhancement (DCE) is the most effective technique to show BBB permeability and reperfusion injury^12^. The aim of this study was to visualize the BBB permeability after reperfusion in hyperglycemic and non-hyperglycemic stroke rats by using multimodel MRI, to verify the effect of hyperglycemia on ischemia-reperfusion injury, and to explore the feasibility of noninvasive observation of ischemia-reperfusion injury in hyperglycemic ischemic stroke using MRI technique.

## Results

**Figure 1:**
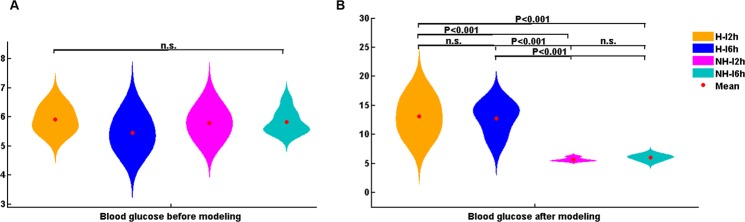
The blood glucose values of all four groups showed in charts (mmol/L). Before diabetes modeling, there were no significant difference between the four groups. After diabetes modeling, the blood glucose increased significantly in hyperglycemia groups compare to non-hyperglycemia groups (*p* < 0.001), which confirmed the successful establishment of diabetes model.

40 rats were divided into 4 groups: hyperglycemic ischemic 2-hr (H-I2h), hyperglycemic ischemic 6-hr (H-I6h), non-hyperglycemic ischemic 2-hr (NH-I2h), and non-hyperglycemic ischemic 6-hr (NH-I6h) groups according to the duration of ischemia and blood glucose levels. Among the 40 rats, 2 rats of the H-I2h, 3 rats of the H-I6h group, and 1 rat of the NH-I6h group died after middle cerebral artery occlusion (MCAO) and were excluded from the study. The study included a total of 34 rats. The blood glucose values of all four groups before and after induction of hyperglycemia are shown in Fig. 1 (mmol/L).

### Effects of Recanalization Time-Point and Blood Glucose on Ischemia-Reperfusion Injury

**Figure 2:**
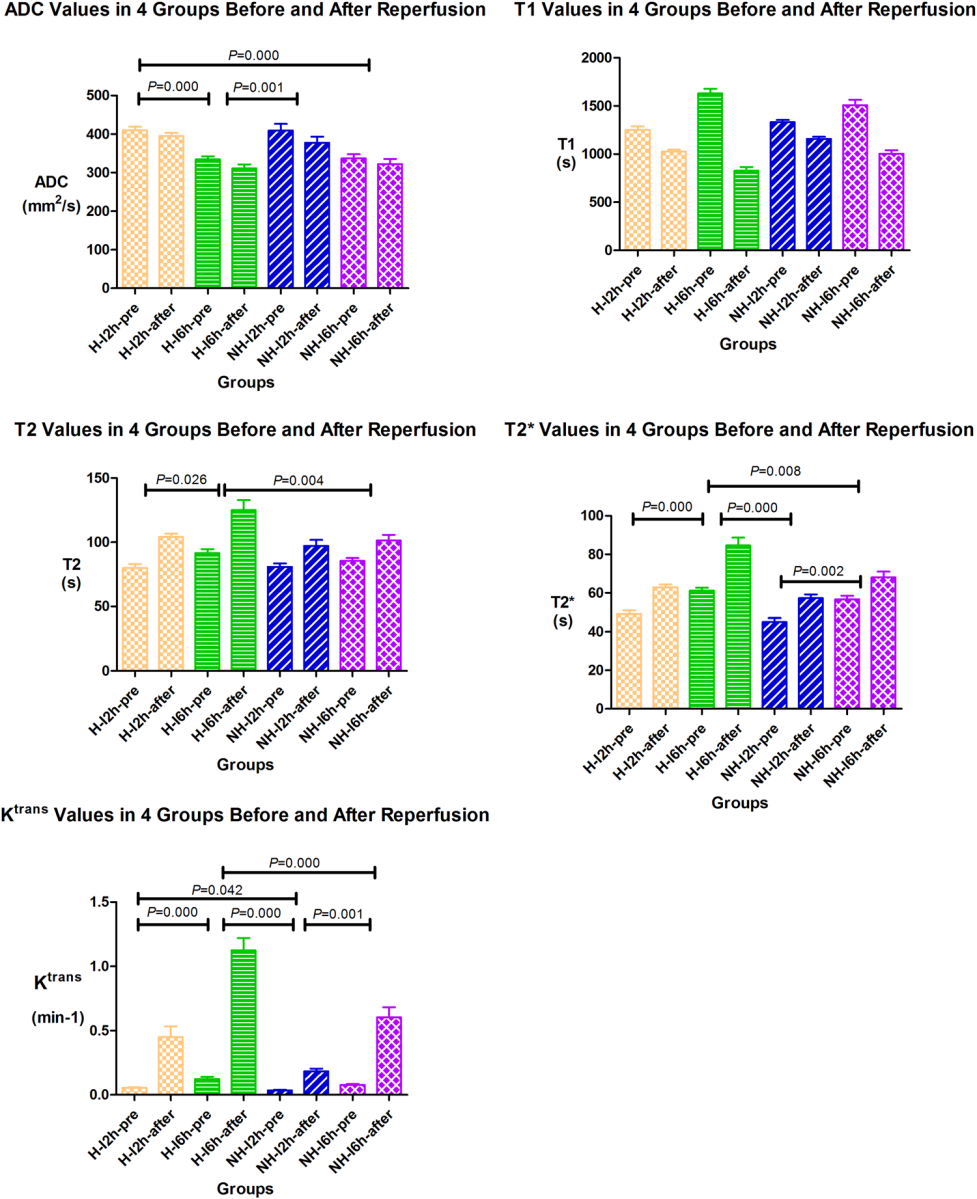
The repeated ANOVA was used to compare parameters before and after reperfusion between groups, with the 4 different groups as between effect, and before/after reperfusion as within effect. ADC, and T1 decreased, T2, T2*, and K^trans^ increased after reperfusion as compared to before reperfusion in all four groups. Post-hoc text was performed between H-2h, H-6h, NH-2h, and NH-6h groups, and the significant results showed in Fig. 2.

T2 weighted (T2W) imaging, diffusion weighted (DW) imaging, T2 mapping, T2* mapping, DCE, and T1 mapping after enhancement sequences were acquired before reperfusion and approximately 3-hr after reperfusion. Apparent diffusion coefficient (ADC), T1, T2, T2*, and K^trans^ values of ischemic lesion were obtained in different groups (see Fig. 2).

**Figure 3:**
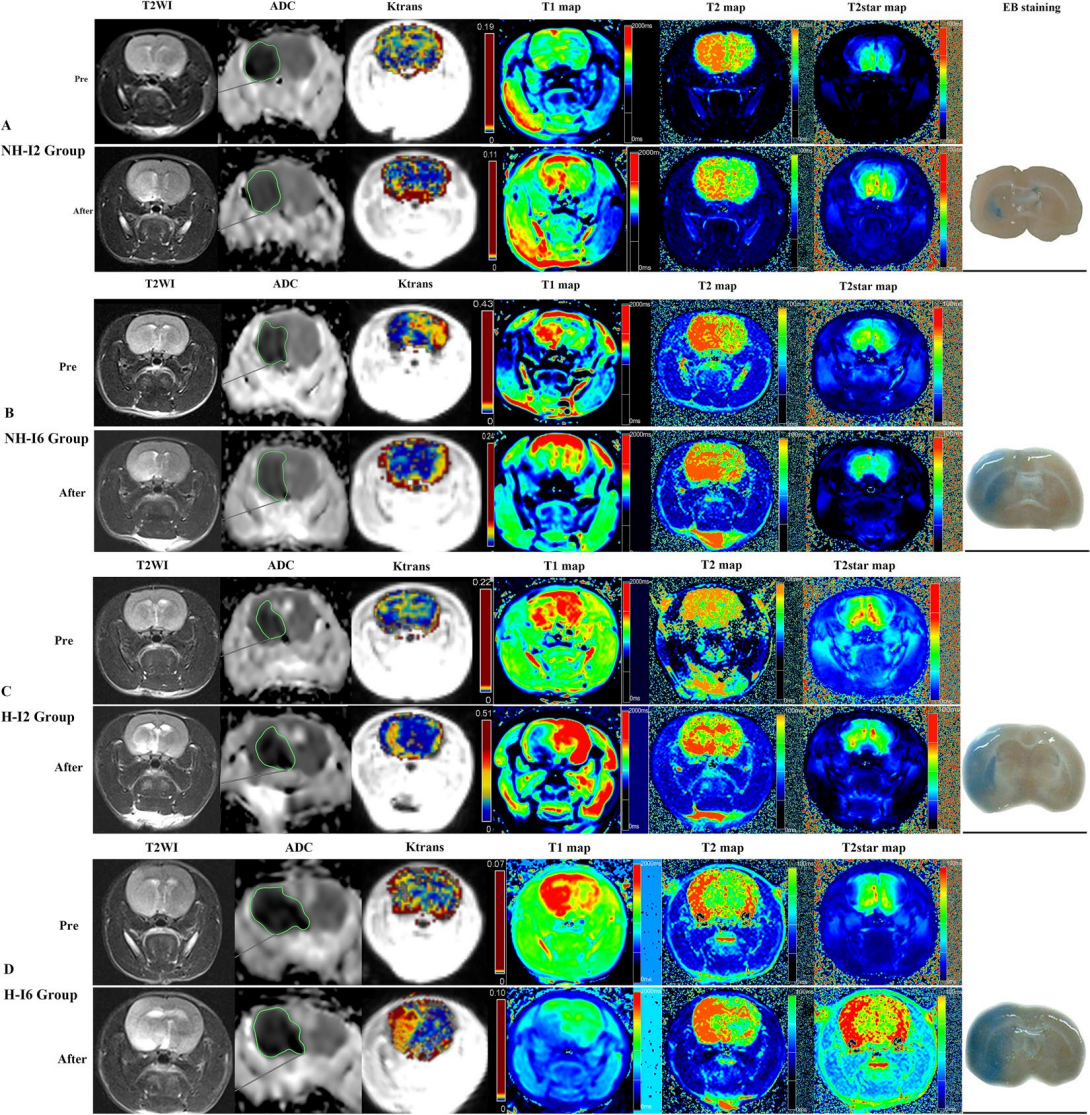
A rat (**A**) belongs to the NH-I2 group. T2WI and ADC showed ischemia of the left middle cerebral artery blood supply area. Recanalization after 2 h of ischemia. ADC (before reperfusion, 410.2; after reperfusion, 427) value increased slightly; T2 (before reperfusion, 83.2; after reperfusion, 95.9), T2* (before reperfusion, 41.5; after reperfusion, 46.2), and K^trans^ (before reperfusion, 0.02; after reperfusion, 0.18) value increased, whereas T1 (before reperfusion, 1381.4; after reperfusion, 1226.2) value decreased slightly after reperfusion. EB staining showed a little blue stain in basal ganglia. A rat (**B**) belongs to the NH-I6 group. T2WI and ADC showed ischemia of the left middle cerebral artery blood supply area. Recanalization after 6 hours of ischemia. T2 (before reperfusion, 80.9; after reperfusion, 86.9), T2* (before reperfusion, 60.1; after reperfusion, 73.4), and K^trans^ (before reperfusion, 0.06; after reperfusion, 0.31) values increased, whereas ADC (before reperfusion, 370.8; after reperfusion, 297) and T1 (before reperfusion 1537.9, after reperfusion, 988.4) values decreased after reperfusion. EB staining showed moderate blue stain in basal ganglia and cortex. A rat (**C**) belongs to H-I2h group. T2WI and ADC showed ischemia of the left middle cerebral artery blood supply area. Recanalization after 2 hours of ischemia. T2 (before reperfusion, 75.4; after reperfusion, 99.3), T2* (before reperfusion, 40.4, after reperfusion, 57.8), and K^trans^ (before reperfusion, 0.05; after reperfusion, 0.3) values increased, whereas ADC (before reperfusion, 387.1; after reperfusion, 384.5) and T1 (before reperfusion, 1237.1; after reperfusion, 1106.6) values decreased slightly after reperfusion. EB staining showed obvious blue staining in basal ganglia. A rat (**D**) belongs to H-I6h group. T2WI and ADC showed ischemia of the left middle cerebral artery blood supply area. Recanalization after 6 hours of ischemia. T2 (before reperfusion, 96.6; after reperfusion, 131.4), T2* (before reperfusion, 66.2; after reperfusion, 85.1), and K^trans^ (before reperfusion, 0.1; after reperfusion, 1.33) values increased, whereas ADC (before reperfusion, 374.1; after reperfusion, 356.7) and T1 (before reperfusion, 1627.2; after reperfusion, 867.5) values decreased after reperfusion. EB staining showed obvious blue staining in basal ganglia and cortex.

The repeated ANOVA was used to compare parameters before and after reperfusion between groups, with the 4 different groups as between effect, and before/after reperfusion as within effect. There was no interaction between grouping and reperfusion in ADC values (*F* = 0.485, *p* = 0.695). ADC decreased after reperfusion as compared to before reperfusion in all four groups (general ischemia/reperfusion effects *p* = 0.002, general group effect *p* < 0.0001). Interestingly, independent of ischemia/reperfusion, ADC values were lower in H-I6h group vs. H-I2h group (post-hoc *p* < 0.0001), lower in H-I6h group vs. NH-I2h group (post-hoc *p* = 0.001), lower in H-I2h vs. NH-I2h group (post-hoc *p* = 1.000), and lower in H-I6h group vs. NH-I6h group (post-hoc *p* = 1.000) (see Fig. 2). Univariate analysis of variance was used to verify the effect of blood glucose level and duration of ischemia (recanalization time-point) on the permeability of BBB, with the difference between before and after reperfusion (e.g. ADC-_difference_ = ADC-_before_ − ADC-_after reperfusion_) of imaging parameters as dependent variable (ADC, T1, T2, T2*, and K^trans^), and the blood glucose level (H vs. NH) and recanalization time-point (2-hr vs. 6-hr) as fixed factors. There is no significant different of ADC-_difference_ values among hyperglycemic group (−18.49 ± 13.94) vs. non-hyperglycemic group groups (−23.98 ± 46.81) (*F* = 0.103, *p* = 0.750) and among groups with different recanalization time-points (2-hr (−23.54 ± 39.28) vs. 6-hr (−18.69 ± 29.81)) (*F* = 0.109, *p* = 0.744) (see Fig. 3).

T1 have significant interaction term effects between grouping and reperfusion (*F* = 79.430, *p* < 0.0001). Post-hoc text was performed between H-I2h, H-I6h, NH-I2h, and NH-I6h groups, and the significant result showed in Fig. 2. The results of univariate analysis of variance showed that the T1 values also decreased after reperfusion as compared to before reperfusion in all four groups, with a higher degree of decrease in hyperglycemic group (−478.74 ± 303.70) than in non-hyperglycemic group (−319.85 ± 197.71) (*F* = 30.887, *p* < 0.0001), with a higher degree of decrease in 6-hr ischemia-reperfusion groups (−643.30 ± 177.53) than 2-hr ischemia-reperfusion groups (−198.29 ± 94.48) (*F* = 203.807, *p* < 0.0001) (see Fig. 3).

T2 have significant interaction term effects between grouping and reperfusion (*F* = 6.054, *p* = 0.002). Post-hoc text was performed between H-I2h, H-I6h, NH-I2h, and NH-I6h groups, and the significant result showed in Fig. 2. The T2 values increased after reperfusion in all four groups, with a higher degree of increase in hyperglycemic group (28.18 ± 9.87) than in non-hyperglycemic group (16.11 ± 9.38) (*F* = 15.442, *p* < 0.0001). However, the T2 values was not obviously different among groups with different recanalization time-points (2-hr (20.02 ± 8.36) vs. 6-hr (24.03 ± 14.13)) (*F* = 1.852, *p* = 0.184) (see Fig. 3).

T2* have significant interaction term effects between grouping and reperfusion (F = 6.220, *p* = 0.002). The significant result of post-hoc text was showed on Fig. 2. The T2* values increased after reperfusion in all four groups, with a higher degree of increase in hyperglycemic group (18.01 ± 8.00) than in non-hyperglycemic group (11.34 ± 5.48) (*F* = 10.402, *p* = 0.003). The change of T2* value was not obviously different among groups with different recanalization time-points (2-hr (12.96 ± 4.93) vs. 6-hr (17.01 ± 9.45)) (*F* = 4.324; *p* = 0.046) (see Fig. 3).

K^trans^ have significant interaction term effects between grouping and reperfusion (*F* = 26.537, *p* < 0.0001). The significant result of post-hoc text was showed on Fig. 2. The K^trans^ values increased after reperfusion in all four groups, with a higher degree of increase in hyperglycemic group (0.66 ± 0.39) than in non-hyperglycemic group(0.31 ± 0.24) (*F* = 28.358; *p* < 0.0001), with a higher increase of decrease in 6-hr (0.75 ± 0.33) than in 2-hr group (0.26 ± 0.21) (*F* = 51.916; *p* < 0.0001) (see Fig. 3).

### Comparison of EB Leakage and MRI Parameters

One-way analysis of variance (ANOVA) showed that differences in evans blue (EB) leakage between the groups were statistically significant (*F* = 55.662; *p* < 0.0001).

The Bonferroni test showed that differences of EB leakage between the groups were statistically significant, excepting H-I2h versus NH-I2h: H-I2h versus H-I6h (*p* < 0.0001); H-I2h versus NH-I2h (*p* = 0.157); H-I2h versus NH-I6h (*p* < 0.0001); and H-I6h versus NH-I6h (*p* = 0.008) (see Fig. 2).

Correlative analysis revealed that EB leakage was positively correlated with T2 values (r = 0.348; *p* = 0.043), T2* values (r = 0.408; *p* = 0.017), and K^trans^ values (r = 0.919; *p* < 0.0001); negatively correlated with T1 values after enhancement (r = −0.8360; *p* < 0.0001). EB leakage was not significantly correlated with ADC values (*p* = 0.647).

Our results showed that hyperglycemia aggravates the destruction of the BBB and ischemia-reperfusion injury. Furthermore, ischemia-reperfusion injury was more serious in the 6-hr ischemia groups than in the 2-hr ischemia groups.

### Discussion

In this study a unilateral (left) MCAO model was established by thread embolism. The thread was removed to prepare the reperfusion model at a different ischemia time. The affected areas include the left cerebral cortex, the striatum, and other subcortical structures; the striatum was the most vulnerable area. To visualize the effect of hyperglycemia on ischemia-reperfusion injury, multimodal MRI was used to dynamically display the changes of the parameters in different blood glucose levels and different times of duration of ischemia group.

In our study, the number of dead animals in the group with hyperglycemic is larger than that in the non-hyperglycemic group, which indirectly confirms the fact that hyperglycemia complicated with ischemic stroke tends to have a poor prognosis. The ADC value is recognized as the most sensitive MRI parameter for the diagnosis of ischemic stroke. The decrease of ADC value in lesion represents the diffusion limitation caused by cytotoxic edema, and the ADC value provides limited information for BBB permeability^13^. The repeated ANOVA results showed that there were significant differences in all parameters (including ADC T1, T2, T2*, and K^trans^) before and after reperfusion. However, only ADC showed there was no interaction between grouping and reperfusion. And ADC had the minimum difference with the maximum *p* value. A likely explanation is that the decrease of ADC value is a result of the aggravation of the cytotoxic edema and the decrease of the diffusivity of infarcted brain tissue as the ischemia time is prolonged. On the other hand, according to previous study, hyperglycemia may influence ADC decline following ischemia, suggesting that mechanisms other than membrane depolarization and cell swelling may contribute to changes in ADC in cerebral ischemia^14^. Univariate analysis of variance showed that the variation of ADC values after reperfusion has no significant difference between hyperglycemic and non-hyperglycemic groups. There is no significant correlation between the ADC value and the leakage of EB staining. The same as in the previous study, it is suggested that ADC value has little effect on BBB permeability and ischemia-reperfusion injury^15^.

Hyperintensity in T2W imaging was generally recognized as representative of vascular edema and BBB destruction, thus reflecting non-recoverable damage in stroke^16^. BBB opening and vasogenic edema exacerbate ischemic injury and may contribute to reperfusion injury^17^. Previous studies have confirmed that the infarct volume on T2W imaging was most close to the pathologic result^18^. Our results showed that variation of T1, T2, T2*, and K^trans^ values after reperfusion has significant difference in all groups, and has interaction between grouping and reperfusion. The T2 value and the T2* value increased in all groups after reperfusion. It was confirmed that the vasogenic edema and reperfusion injury was aggravated with the prolonged ischemia time^19,20^. The increase was more obvious in the hyperglycemic group than in the non-hyperglycemic group and more obvious in the ischemic 6-hr group than in the 2-hr ischemic group. This is yet another example demonstrating that hyperglycemia aggravates ischemia-reperfusion injury. Shen *et al*.^21^ document T2 and BBB permeability have the different peak times, which suggest that at least some of the factors responsible for BBB permeability increase differ from those responsible for edema. In this study, The T2 and T2* values also were moderate positively correlated with the leakage of EB staining. It is suggested that, to a certain extent, T2 and T2* values can reflect the BBB permeability of ischemic stroke. The possible mechanism is that the vasogenic edema and the increased permeability of BBB, caused increases and retention the water content of ischemic brain tissue, and then caused increased T2 and T2* values. However, the correlation coefficient (r) is less than 0.5, and the correlation is not close.

Previous studies have shown that delayed T1 enhancement is a potential method that can provide temporal and spatial changes of BBB and the evaluation of the development of cerebral ischemia reperfusion^22^. The most common contrast agent in MRI is gadolinium diethylenetriaminepentaacetic acid (Gd-DTPA), which contains 7 unpaired electrons with very strong paramagnetism, which can obviously shorten the time of T1 of tissue. Therefore, enhanced lesion on the delayed T1 enhancement is caused by the accumulation of Gd-DTPA in the extracellular space, changing the relaxation time of the proton in the adjacent tissue. Gd-DTPA cannot pass the BBB of normal brain tissue, and reperfusion injury leads to BBB damage. Only BBB damage can cause the contrast agent to leak out into the brain, resulting in the shortened Tl time and the increased T1W imaging signal such as reperfusion injury in stroke^23^. However, this method needs to be scanned for a certain time after the enhancement of the scan to get the degree of enhancement after the delayed enhancement; delayed T1W imaging scan is difficult to use in clinical practice. DCE is a functional imaging technology based on T1W imaging-enhanced scanning technology. DCE is currently the best imaging method for noninvasive evaluation of BBB *in vivo*^24,25^. DCE MRI permits quantitative evaluation of the leakage of contrast agents across the BBB, most frequently via the blood-to-brain transfer constant, K^trans^, of Gd-DTPA. K^trans^ = E×F, where F is the plasma flow and E is the extraction fraction^25^. Our results confirm that the delayed T1 and K^trans^ values can reflect the BBB permeability and that T1 has a strong negative correlation with EB. K^trans^ has a higher positive correlation with EB leakage compared with other parameters. The sensitivity of the K^trans^ value change is higher than that of the T1 value. Guangliang Ding *et al*.^26^ also document among T1, T2, K^trans^ and ADC value, K^trans^ was the powerful marker for BBB permeability. The results of our study showed that the increased K^trans^ value and the decreased T1 value in the 6-hr group were more obvious than in the ischemic 2-hr group; that is, the reperfusion injury is more obvious. This is the same as the previous research results^27^; the longer the duration of ischemia, the more serious the reperfusion injury is. As the duration of ischemia is prolonged, the greater the degree of BBB permeability increases after reperfusion, the shorter the time it takes to heal, the more serious the ischemia-reperfusion injury, which is a long time of ischemia and recanalization.

At the same time and in the same duration of ischemia, reperfusion injury in the hyperglycemic group was more serious than in the non-hyperglycemic group, confirming that hyperglycemia aggravated the endothelial injury and reperfusion injury in the ischemic brain tissue. The severity of reperfusion injury in our study, sorted according to K^trans^ value of four groups, is the H-I6h group > the NH-I6h group > the H-I2h group > the NH-I2h group.

Multimodel MRI could noninvasively reveal visible BBB permeability after ischemic stroke. Among the several parameters, the ADC values was not significantly correlated with EB leakage, therefor ADC could provide limited information of BBB permeability. However, T1, T2, T2*, and K^trans^ from DCE were significantly correlated with EB leakage, then could provide information of BBB permeability. Zamir Merali *et al*.^24^ documented that BBB permeability may be most elevated at 6–48 hrs after stroke onset. The permeability of BBB in ischemia 6-hr is higher than in ischemia 2-hr group. Our results showed that T1 and K^trans^ were different in 2-hr ischemia groups and 6-hr ischemia groups, while ADC, T2, and T2* were similar in 2-hr and 6-hr ischemia groups. And K^trans^ have the best ability to distinguished between groups based on multi multiple comparison. Based on these results, K^trans^ should be the best imaging marker for ischemia-reperfusion injury in hyperglycemic rats.

Limitation: A limitation of our study is the manual selection of region of interests (ROIs). To reduce inter-observer variability, the same investigator placed all ROIs during the experiment. Another limitation is the effect of hyperglycemia on cerebrovascular disease, which is a chronic disease. Hyperglycemic rat models experience only a short period of hyperglycemia. Further translational medicine should be made to confirm our results.

## Material and Methods

This study was performed according to the National Institutes of Health guidelines for the care and use of laboratory animals. All protocols were approved by the medical experiment animal administrative committee of Hainan General Hospital.

### Animal Model and Experimental Protocol

In this study, 40 healthy adult male Sprague-Dawley (SD) rats (180~230 g, 6~7-week age) from the Hainan Medical College were used. The rats were anesthetized using halothane (3.5% induction, 0.7% to 1.5% maintenance) in a 2:1 mixture of N_2_O/O_2_ and the core temperature was maintained at 36 to 37 °C throughout all surgical and MRI procedures.

Considering the duration of ischemia and blood glucose levels, the animals were divided into H-I2h, H-I6h, NH-I2h and NH-I6h group.

Hyperglycemia was induced in SD rats of hyperglycemic groups by feeding them a high-fat diet (40% of calories from fat) for 2 weeks. Then, a single intraperitoneal dose (35 mg/kg) of streptozotocin (Zanosar, Sigma Chemical Co, St. Louis, MO), a naturally occurring chemical that is particularly toxic to the insulin-producing beta cells of the pancreas in mammals, was injected and the high-fat diet was continued for another 2 weeks. The blood glucose level was measured twice (before and after 4 weeks high-fat diet), using test strips for glucose (Polymer Technology System, Indianapolis, IN) to confirm hyperglycemia.

MCAO was produced using an adaptation of a model developed by Zea Longa *et al*.^28^. Briefly, after a midline skin incision was made, the right common internal and external carotid arteries were exposed. Then the right MCAO was performed by the intraluminal insertion of a special MCAO monofilament for rats (Beijing Sunbio Biotech Company, Beijing) from the right external carotid artery into the right internal carotid artery until it blocked the origin of the middle cerebral artery (MCA) (19 to 21 mm). Choosing the size of the monofilament (a diameter of 0.34 to 0.38 mm) depends on the patient’s body weight. Reperfusion was initiated at 2 hours for the H-I2/NH-I2 groups and 6 hours for the H-I6/NH-I6h group through removal of the thread and tying off the distal external carotid artery^29^.

### MRI Protocol

All MRI scans were acquired on a clinical whole-body 3.0 Tesla scanner, MRI (Verio, Siemens Medical Solutions, Erlangen, Germany), with an 8-channel special animal coil. Serial images of the brain were acquired before reperfusion and approximately 3-hr after reperfusion^30^. Ischemia-reperfusion for all rats. T2W imaging, DW imaging, T2 mapping, T2* mapping, DCE, and T1W imaging after enhancement sequences were performed. For each imaging sequence, the parameters were designed to provide the same slice thickness (2 mm), slice gap (0 mm), field of view (FOV, 64 × 64 mm^2^), and matrix (256 × 256 mm^2^).

T2W imaging scans (repetition time/echo time [TR/TE] = 3500 ms/89 ms; number of excitations [NEX] = 5) were obtained using a fast spin-echo sequence. Scanning time was 1 min 30 s.

DW imaging scans (TR/TE = 4000 ms/83 ms) were obtained using a two-dimensional spin-echo echo planar imaging (SE-EPI) sequence. ADC maps were generated from 3-point analysis based on 3 different b values (0, 500, and 1000 s/mm^2^). Scanning time was 3 min 30 s.

The MapIt sequence was used with five echo scans for T2 mapping. The parameters were as follows: TR, 800 ms; TE, 11.8, 23.6, 35.4, 47.2, 59 ms; FA, 180°; average, 2; scanning time, 5 min 30 s.

The MapIt sequence was used with five echo scans for the T2* mapping. The parameters were as follows: TR, 445 ms; TE, 4.4, 11.9, 19.7, 27.3, 34.8 ms; FA, 60; average, 2; scanning time, 5 min 43 s.

The DCE-MRI was performed using the following sequences. Two pre-contrast data sets were acquired by T1-vibe (TR/TE, 5.39/2.15 ms) with flip angles of 5° and 15°. This was followed by a DCE acquisition series using T1-twist (TR/TE, 5.39/2.15 ms) with a flip angle of 12°, which consisted of 60 measurements with a temporal spacing of 4.29 seconds. After the fifth baseline acquisition, a Gadolinium (Gd)-based contrast agent (Gd-DTPA; Omniscan, GE Healthcare, Oslo, Norway) was injected through the femoral vein at a dose of 0.1 mmol/kg of body weight. Scanning time was 5 min 23 s.

The T1 FLIP sequence was used for T1 mapping. T1 mapping was acquired after DCE. The parameters were as follows: TR, 15 ms; TE, 3.5 ms; FA, 5, 26; average, 5; scanning time, 6 min 7 s.

### MRI Analysis

Post-processing analysis was performed using workstation software (Syngo Tissue 4D). The Tofts and Kermode pharmacokinetic model was used to model the relationship between the tissue concentration of the contrast agent and the blood concentration time curve of the contrast agent. Arterial input functions (AIF) was automatically selected by software. The whole rat brain was outlined as the region of counting parameters to generate the whole brain volume transfer constant (K^trans^) map.

Areas of ischemia were identified as regions of reduced diffusion relative to a normal cortex on ADC maps and were the basis for the ROI selection. The ROIs of the ADC maps were saved and later copied onto the K^trans^ map, T2, T2*, and T1 map, and the K^trans^ value, average ADC value, T2, T2*, T1 relaxation time (ms), and standard deviation values were calculated.

### Histologic Staining (Evans Blue Staining)

After MRI all rats were processed for EB staining. In brief, 2% EB solution (4 mL/kg) was infused through the femoral vein and a 16-gauge needle was inserted through the left ventricle an hour later. The left atrial appendage was cut open and flushed with saline until the liquid from the left atrium became colorless. The brain was cut into 2-mm thick slices and photographed. The weighed hemispheres were placed in 50% trichloroacetic acid, homogenized before centrifugation for 20 min (10,000 r/m), and the supernatant was diluted 1:2 with diethyl ether followed by fluorescence spectrophotometry at λ = 620 nm. The curve was used to determine the content of EB in the brain and the amount of EB in both cerebral hemispheres.

### Data and Statistical Analysis

SPSS for MAC (Version 22; IBM SPSS) was used for data analysis. The repeated measures ANOVA was used to compare parameters values before and after reperfusion between groups, with the 4 different groups as between factor, and before/after reperfusion as within factor. A Univariate analysis of variance of the general linear model was used to verify the effect of blood glucose level and duration of ischemia (recanalization time-point) on the permeability of BBB. The one-way ANOVA was used to compare the blood glucose the EB staining leakage with that of different groups. Multiple comparisons were performed using Bonferroni test. The correlation of the EB staining leakage and imaging parameters values was analyzed by Pearson correlation. A *p* < 0.05 was considered statistically significant.

## Conclusion

Multimodal MRI was used to monitor ischemia-reperfusion injury in diabetic rats. The T2, T2*, T1, and K^trans^ values all can reflect the BBB permeability to some extent, thus visualizing the reperfusion injury. Among them, the K^trans^ values may be the best MRI parameter reflecting the BBB permeability. This study confirms that hyperglycemia aggravates ischemia-reperfusion injury and may be an important risk factor for the prognosis of ischemic stroke.
